# Semisolid Microstructural Evolution during Partial Remelting of a Bulk Alloy Prepared by Cold Pressing of the Ti-Al-2024Al Powder Mixture

**DOI:** 10.3390/ma9030199

**Published:** 2016-03-16

**Authors:** Yahong Qin, Tijun Chen, Yingjun Wang, Xuezheng Zhang, Pubo Li

**Affiliations:** State Key Laboratory of Advanced Processing and Recycling of Nonferrous Metals, Lanzhou University of Technology, Lanzhou 730050, China; qyh420@126.com (Y.Q.); wangyingjun@163.com (Y.W.); zhangxz1991@163.com (X.Z.); lipubogs@163.com (P.L.)

**Keywords:** microstructural evolution, Al_p_-Ti_p_-2024Al_p_ bulk alloy, powder thixoforming, partial remelting, *in situ*, Al_3_Ti phase, stress calculating, Al-Ti diffusion reaction

## Abstract

A new method, powder thixoforming, has been proposed to fabricate an *in situ* Al_3_Ti_p_/2024Al composite. During partial remelting, the microstructural evolution of the bulk alloy prepared by cold pressing of the Ti, Al, 2024Al powder mixture was investigated, and the formation mechanism of the Al_3_Ti particles produced by the reaction between the Ti powder and the Al alloy melt is also discussed in detail. The results indicate that the microstructural evolution of the 2024 alloy matrix can be divided into three stages: a rapid coarsening of the powder grains; a formation of primary α-Al particles surrounded with a continuous liquid film; and a slight coarsening of the primary α-Al particles. Simultaneously, a reaction layer of Al_3_Ti can be formed on the Ti powder surface when the bulk is heated for 10 min at 640 °C The thickness (*X*) of the reaction layer increases with the time according to the parabolic law of $X=-0.43t^{2}+4.21t+0.17$. The stress generated in the reaction layer due to the volume dilatation can be calculated by using the equation $\sigma_{Al_{3}Ti}=-\frac{E_{Al_{3}Ti}}{6\left(1-\upsilon_{Al_{3}Ti}\right)}\frac{{t^{2}}_{Al_{3}Ti}}{t_{Ti}}\left(\frac{1}{R}-\frac{1}{R_{0}}\right)$. Comparing the obtained data with the results of the drip experiment, the reaction rate for the Ti powder and Al powder mixture is greater than that for the Ti plate and Al alloy mixture, respectively.

## 1. Introduction

Particle-reinforced aluminum matrix composites have been widely used in the automobile, aviation, and aerospace industries due to their valuable properties. Over the past few decades, ceramic particle-reinforced aluminum matrix composites have attracted a large number of researchers [1,2,3]. Ceramic particles such as SiC, Al_2_O_3_, and TiB_2_ are used as reinforcements to fabricate particle-reinforced aluminum matrix composites [1]. However, the poor wettability and the significant mismatch in the thermal expansion coefficient (CET) between the aluminum matrix and the ceramic reinforcements result in a large stress concentration at the reinforcement/matrix interfaces, which degrades the mechanical properties of the composites. At the same time, intermetallics, such as Al_3_Ti [4], Al_3_Ni [5], and Al_3_Fe [6], have CET values comparable with that of the Al matrix (besides high modulus and strength). In addition, the interfacial bonding strength is greater if the reinforcements are generated *in situ*. Among them, the Al_3_Ti is the most commonly used in Al matrix composites because of its low density, high Young’s modulus, and good wettability with an aluminum matrix [7,8,9,10]. More important, it can be easily generated through an *in situ* reaction between Al and Ti at high temperature.

Several manufacturing techniques have been recently developed to fabricate the Al_3_Ti particle (Al_3_Ti_p_)-reinforced Al matrix composites, such as powder metallurgy, centrifugal solid-particle method, mechanical alloying, and an *in situ* casting technique [11,12,13]. Generally, powder metallurgy is considered a prominent method for commercial production of aluminum matrix composites due to a uniform distribution of reinforcements and flexible constituent design [14,15]. It consists of a mixture of the aluminum powder with titanium or titanium dioxide powder sintered at a temperature below the solidus temperature of the aluminum powder with or without pressure [11,12]. However, this technique has many disadvantages such as long reaction time, high energy consumption, low productivity, and difficulty to produce large-sized parts with complicated shapes [12]. In particular, a pore formation due to the Kirkendall effect results in a decreased density of the composite.

The thixoforming method is able to significantly decrease or even eliminate porosities and thus produce materials with compact microstructures. Furthermore, it is also valid for manufacturing large-sized components which also have complex shapes [16]. The existing studies show that a semisolid microstructure with small and nearly spherical particles can be obtained after the Al alloy bulk which is prepared by cold pressing the alloy powders that are partially remelted [17,18]. Therefore, a new method of making Al_3_Ti_p_/Al matrix composites, powder thixoforming, can be proposed by combining powder metallurgy with thixoforming. The Ti and Al alloy powders are initially cast into the starting ingots for thixoforming by using the ball-milling and cold-pressing procedures of powder metallurgy. Then the ingots are partially remelted and thixoformed. After being partially remelted, a semisolid microstructure with small and spheroidal primary particles can be produced, and, most significantly, Al_3_Ti particles can be generated through the *in situ* reaction between the Ti powder and the Al in the liquid phase. It is well known that obtaining an ideal semisolid microstructure is the key to the thixoforming technology. To obtain this desirable microstructure, we must investigate the microstructural evolution of *in situ* composites during the partial remelting stage. However, there are as of yet no investigations on the microstructural evolution of *in situ* Al_3_Ti_p_/Al matrix composites fabricated by the powder thixoforming technology in the literature. The existing research efforts are only focused on the microstructural evolution during partial remelting of artificial composites’ cold-pressed bulk prepared by cold pressing the mixed powders, such as the SiC_p_/Al composites [17,18]. 

Therefore, it is necessary to verify the microstructural evolution during partial remelting in order to determine the optimum process conditions for fabricating *in situ* Al_3_Ti_p_/Al composites. Because the partial remelting stage is the most important stage of the whole process, it defines not only the semisolid microstructure, but also the formation mechanism of the Al_3_Ti reinforcements. Therefore, in this research work, the microstructural evolution, and especially the formation mechanism of the Al_3_Ti particles through the *in situ* reaction, were investigated during partial remelting of the alloy bulk which is prepared by cold pressing the mixed pure aluminum powder (Al_p_), pure titanium powder (Ti_p_), and 2024 aluminum alloy powder (2024 Al_p_).

## 2. Experimental

**Figure 1:**
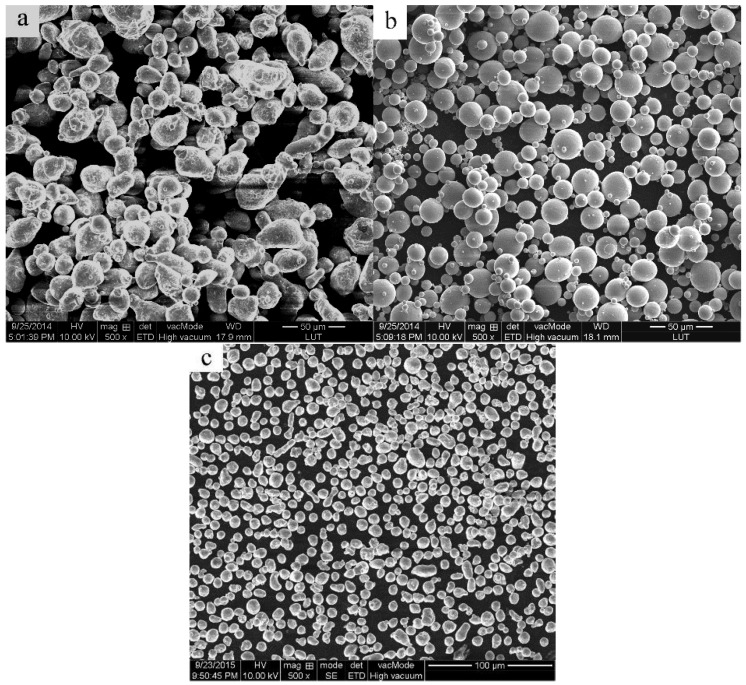
SEM micrographs of the as-received powders. (**a**) 2024 Al_p_; (**b**) Ti_p_; (**c**) Al_p_.

**Figure 2:**
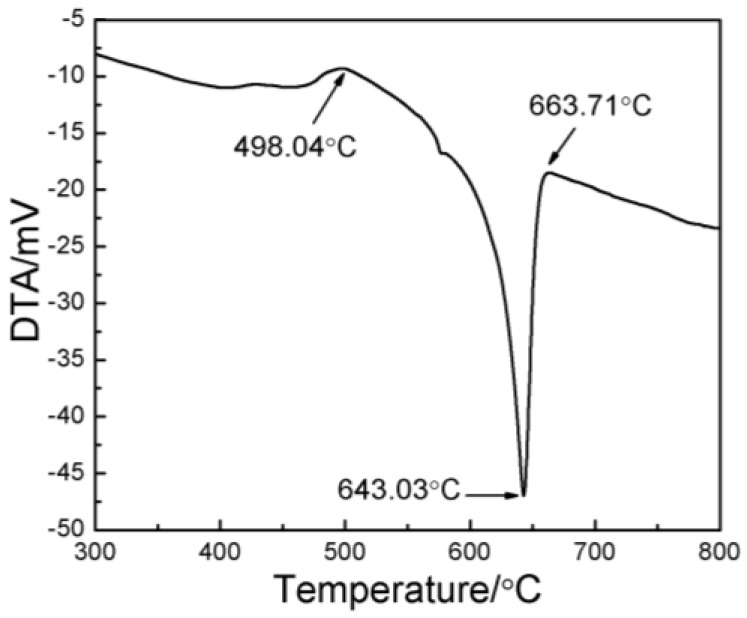
A DTA curve for 2024 Al_p_.

The materials used in this work were 2024 Al_p_, Al_p_, and Ti_p_ produced by atomization. The nominal chemical composition of 2024 Al_p_ includes 4.22% Cu, 1.35% Mg, 0.5% Si, 0.5% Fe, 0.5% Mn, and a balance of Al (in wt.%). The average size of the 2024 Al_p_ powder is 20.568 μm. The Al_p_ powder has a purity of 99.8% and an average size of 11.817 μm, while the Ti_p_ powder has a purity of 99.2% and an average size of 17.197 μm. All the powders in this study have a spherical morphology (Figure 1). Because the reaction between the Al and Ti powders consumes some Al, an additional amount of Al_p_ was introduced according to the molar ratio of Al:Ti = 3:1 in order to maintain the composition of the 2024 Al_p_ matrix. In accordance with the composition of the 10 vol.% Al_3_Ti_p_/2024Al composite, 52.71 g of 2024 Al_p_, 4.58 g of Al_p_, and 2.71 g of Ti_p_ were blended inside a ND7-21 planetary ball-milling machine (Nanjing Levinstep Technology Co., Ltd., Nanjing, China). The utilized ball-to-powder weight ratio, rotation speed, and milling time were 5:1, 100 rpm, and 40 min, respectively. To measure the partial remelting temperature, a Pyris Diamond TG/DTA (thermal gravity/differential thermal analyzer) differential thermal analyzer (DTA, NETZSCH, Bavaria, Germany) was used to study the solidification range of 2024 Al_p_; and the obtained solidus and liquidus temperatures were 498.04 °C and 663.71 °C, respectively (Figure 2).

The mixed powders were cold pressed into billets with dimensions of Φ 22 mm × 5 mm using a XH-300KN pressure machine (Tianjin Xingheng Instrument Factory, Tianjin, China). The utilized pressure and holding time values were 180 MPa and 5 min, respectively. A number of billets were produced by repeating the experimental procedure described above. Some of them were heated afterwards in a vacuum resistance furnace for various lengths of time (0−210 min) at 640 °C to study the microstructural evolution. All of the heated specimens were quickly water-quenched and then cut into two parts along the radial direction. A cross-section of each specimen was ground using waterproof abrasive paper before being polished. The specimens were then etched by a 10% NaOH aqueous solution and analyzed with a scanning electron microscope (SEM; FEI, Hillsboro, OR, USA) equipped with an energy dispersive spectrometer (EDS) and an optical microscope (OM; Nikon Instruments, Shanghai, China). The liquid and solid fractions, primary particle sizes, and a thickness of the Al_3_Ti reaction layer were examined by the Image Plus Pro software (Media Cybernetics Company, Silver Spring, MD, USA). The phase constituents were analyzed by X-ray diffraction (XRD; Rigaku, Tokyo, Japan).

## 3. Results and Discussion

### 3.1. Microstructure of the Cold-Pressed Ingots

**Figure 3:**
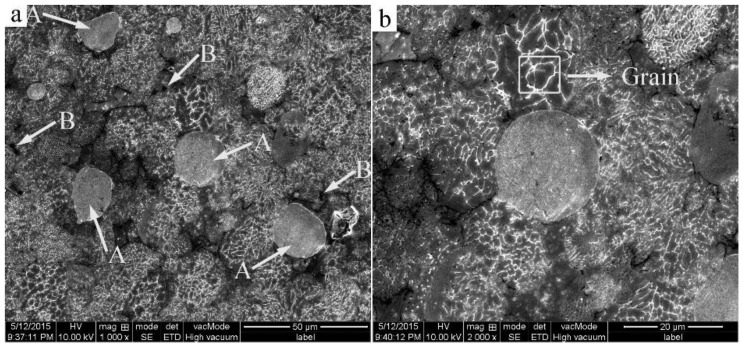
SEM micrographs of the cold-pressed bulk alloy. (**a**) Low magnification; (**b**) High magnification.

In order to clarify the microstructural evolution during partial remelting, the initial microstructure of the cold-pressed ingots should be studied first. The Ti particles (marked by arrows A in Figure 3) are uniformly distributed in the powder matrix. In addition, small pores exist between the particles as shown by arrows B indicating that the microstructure of the cold-pressed bulk alloy is not compact. The grains of the 2024 Al powder are very fine, and the white eutectics are discontinuously distributed in the interdendritic regions.

### 3.2. Microstructural Evolution of the 2024 Alloy Matrix during Partial Remelting

**Figure 4:**
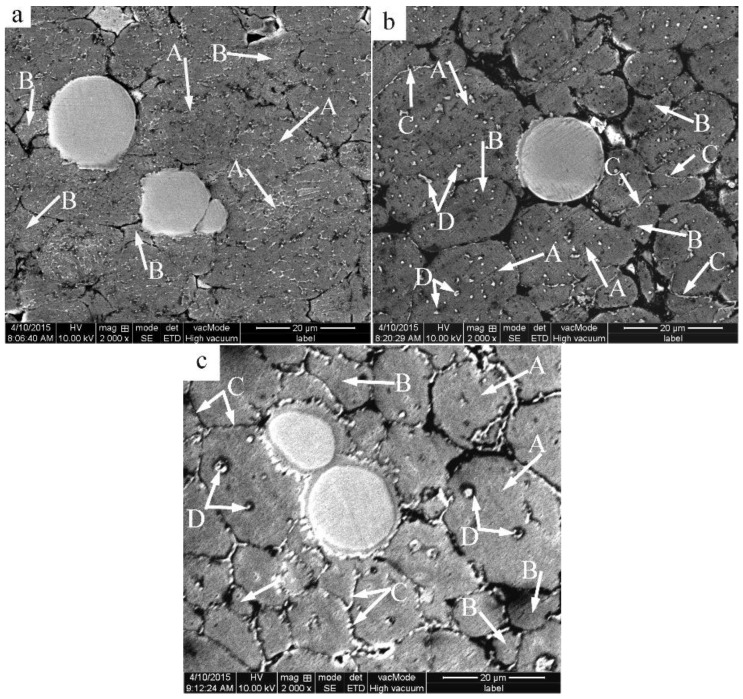
SEM micrographs of the mixed powder bulk alloy heated at 640 °C for different time lengths and then water-quenched. (**a**) 5 min; (**b**) 10 min; (**c**) 15 min.

**Figure 5:**
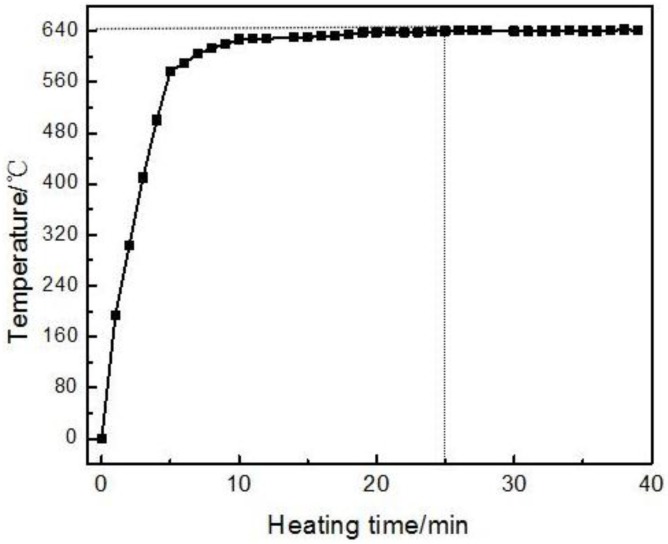
Temperature variations in the specimen with heating time.

Figure 4 presents the microstructures of the specimens heated at a semisolid temperature of 640 °C for different lengths of time. The number of white eutectics in the 2024 Al powder gradually decreases with time during heating. When the heating time reaches 5 min, only few eutectics are left, and the grain boundaries almost disappear (Figure 3b and Figure 4a). This phenomenon observed for other as-cast alloys indicates that the dissolution of the eutectics surrounding the primary particles results in the coarsening of the fine α-Al grains [19,20]. This coarsening mechanism is equally applicable to the present study. The primary particles also coarsen via merging due to their small size (about 2.3 μm). Thus, it can be concluded that the main phenomenon occurring during the first 5 min of heating is eutectics dissolution and coarsening of the small grains. As the heating time reaches 10 min, the eutectics no longer have enough time to completely dissolve in the grains due to a rapid temperature rising (Figure 5). When the temperature reaches the eutectic point, the residual eutectics melt away forming many small liquid phase pools (marked by arrows D in Figure 4b) inside the particles accompanied by the disappearance of the grain boundaries. The original particles thus evolve into compact and spheroidal primary α-Al particles with many liquid pools (Figure 4b). Simultaneously, some discontinuous liquid layers (marked by arrows C in Figure 4b) are formed due to the residual eutectics and melting of some pure and small 2024 aluminum particles. The composition variations in pure aluminum (marked by arrows B in Figure 4) and 2024 aluminum (marked by arrows A in Figure 4) are listed in Table 1. It indicates that the solute elements (such as Cu and Mg) concentrate in the 2024 Al (especially in its liquid phase) and diffuse into the pure aluminum resulting in an alloy formation. The alloying process makes the pure aluminum powder form spherical solid solutions with compositions similar to those for the 2024 Al powder. As the heating time reaches 15 min, the number of the liquid pools (marked by arrows D in Figure 4c) within the particles decreases, and their size increases, which can be explained by the liquid pools merging due to a decrease in the liquid/solid interfacial energy. The discontinuous liquid layers transform into the continuous liquid layers surrounding the primary particles (marked by arrows C in Figure 4c) due to the further particle remelting at higher temperatures (Figure 5). Thus, the original structure evolves into a semisolid microstructure with individual spheroidal primary particles separated by a liquid phase. It can be concluded that the formation of the spherical primary α-Al particles and the continuous liquid film around them is the main event occurring after 5−15 min of heating. Furthermore, it can be observed that each spherical particle evolves into a corresponding spherical primary particle of the semisolid microstructure.

**Figure 6:**
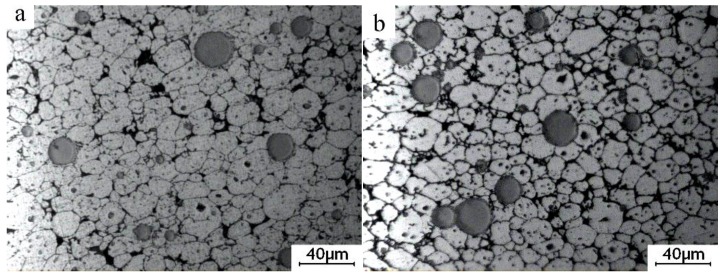

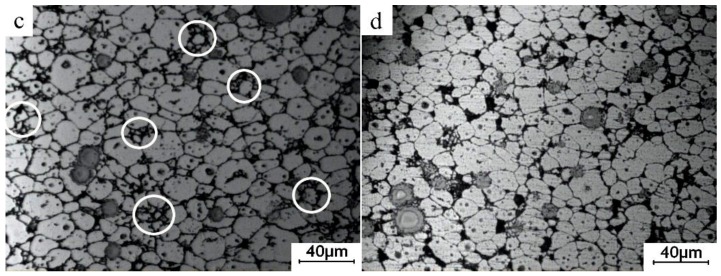
OM micrographs of the mixed powder bulk alloy heated at 640 °C for different lengths of time and then water-quenched. (**a**) 15 min; (**b**) 25 min; (**c**) 60 min; (**d**) 210 min.

**Figure 7:**
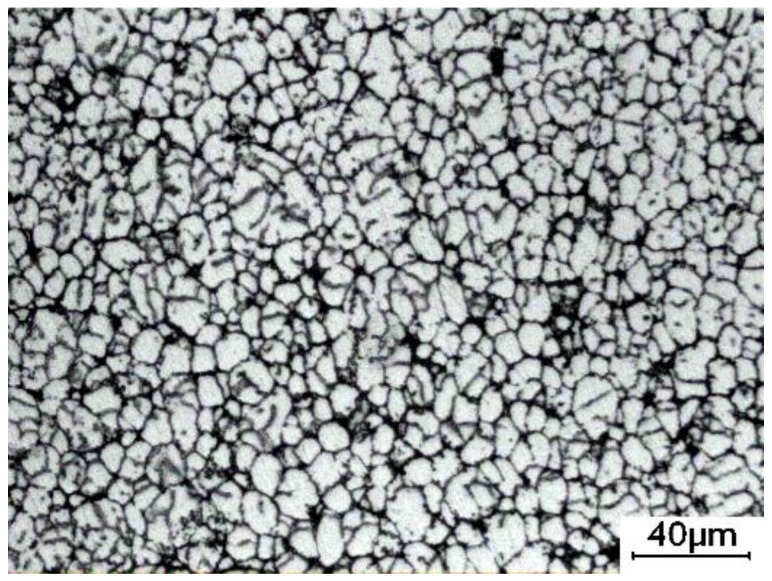
A micrograph of the bulk alloy heated at 750 °C for 60 min and then water-quenched.

**Figure 8:**
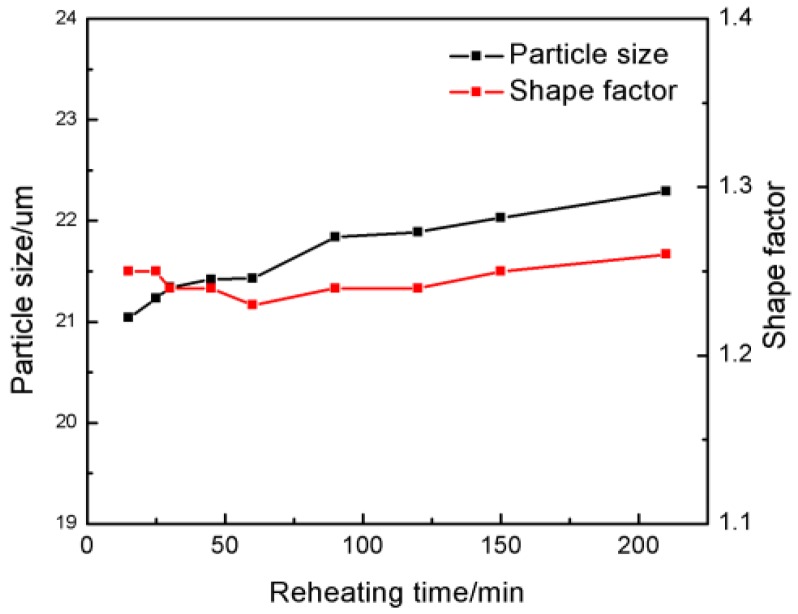
Variations in the primary particle size and shape factor of the bulk alloy with heating time after heating for 15 min at 640 °C.

Figure 6 depicts the microstructure of the bulk alloy heated at 640 °C for over 15 min. Large particles are distributed over the whole structure (Figure 6a), and all the particles are separated only by a thin liquid film (Figure 4c). As the heating time increases, the microstructures are found to consist of small and large particles. The number of the small particles increases with time, while the number of the large particles decreases (comparing Figure 6a–c). It can be suggested that the amount of the liquid phase around the primary particles continuously increases because the primary particles are further partially remelted due to the temperature rising (Figure 5). The liquid phase solidifies into the secondary primary α-Al phase and eutectics during the water quenching. However, the solidification of the liquid phase is very rapid during quenching, so the secondary primary solidified particles are relatively smaller than the primary α-Al particles. Thus, the relatively small particles in the quenched microstructure originate from the solidification of the liquid phase. Figure 7 shows the microstructure of the bulk alloy with a full-liquid 2024 alloy matrix (the bulk alloy was heated for 60 min at 750 °C) after being water-quenched. It shows that this microstructure is composed of small particles and intergranular eutectics. The quantitative examination results indicate that the size of the small particles is about 6 μm. Therefore, the particles less than 6 μm in size can be regarded as a liquid phase. From this standpoint, Figure 6c clearly shows that the liquid phase (marked by circles in the Figure 6c) is evenly distributed between the particles. Moreover, the shapes of the primary particles in the present microstructure are always spherical because the shapes of the initial particles are also spherical. A slight coarsening of the spherical primary particles occurs in order to decrease the solid/liquid interfacial energy, which is the main event during this stage (Figure 8). As the heating time further increases, the solid-liquid two-phase system achieves a dynamic equilibrium when the liquid amount is maintained at a constant value. However, the shape of the primary particles becomes slightly irregular after heating for 210 min (Figure 6d). The diffusion reaction between Al and Ti may be suggested to have an influence on the microstructural evolution of the matrix alloy.

According to Figure 4, a liquid film is formed between the matrix powders with an increase in the remelting time, which results in the reaction between the Al and Ti phases. A discontinuous liquid layer is formed after heating for 5 min due to the partial remelting of the matrix powders. When the heating time is increased, the degree of the powder partial remelting also increases, and the discontinuous liquid layer gradually transforms into a continuous liquid layer because of the higher temperature. The reaction between the Al and Ti phases occurring after 5 min can be explained by the evolution of the liquid film.

Thus, the microstructural evolution of the 2024 alloy matrix can be divided into three stages: a rapid coarsening of powder grains due to the dissolution of the intergranular eutectics (0–5 min), a formation of spherical primary α-Al particles surrounded by a continuous liquid film due to the rapid temperature rising and partial remelting of the powders (5–15 min), and a slight coarsening of the spherical primary α-Al particles in order to reduce the interfacial energy (after 15 min).

### 3.3. Formation of the Al_3_Ti Particles

**Figure 9:**
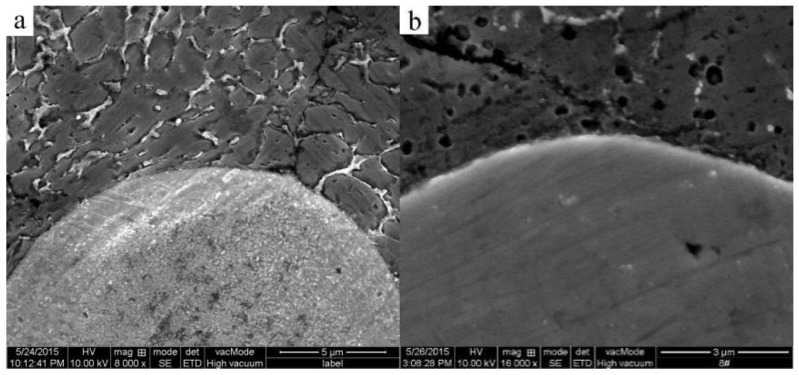

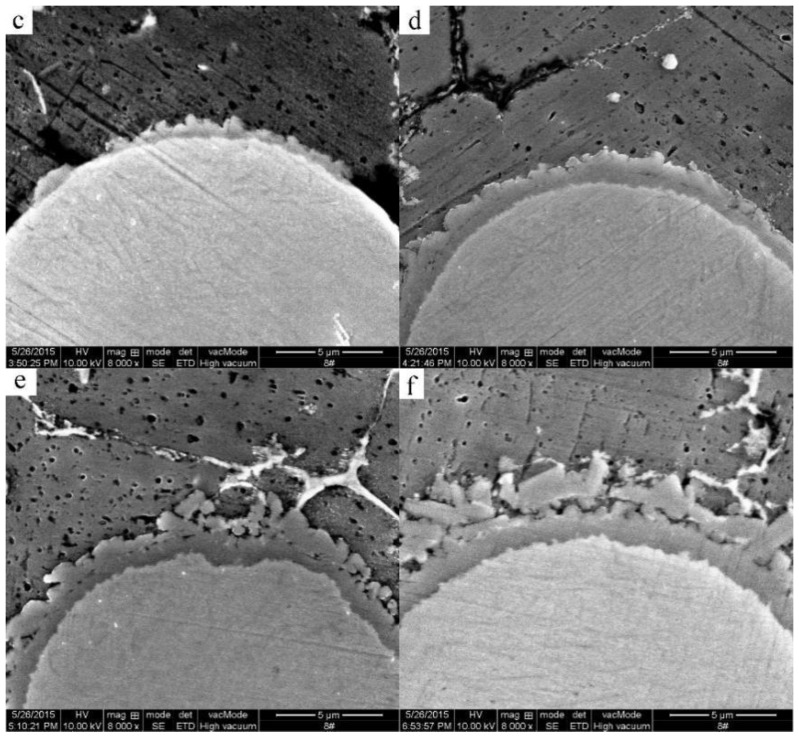
High magnification SEM micrographs of the mixed powder bulk heated at 640 °C for different times. (**a**) 0 min; (**b**) 5 min; (**c**) 10 min; (**d**) 15 min; (**e**) 25 min; (**f**) 45 min.

**Figure 10:**
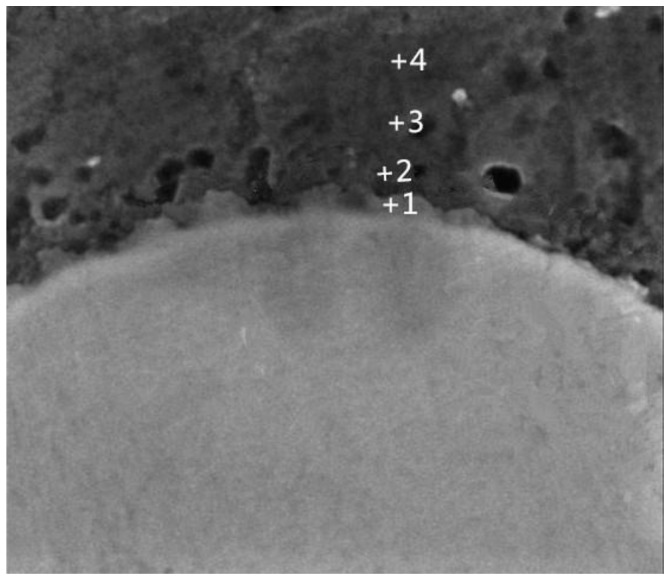
An interface point scanning micrograph of the mixed powder bulk after heating for 5 min at 640 °C.

**Figure 11:**
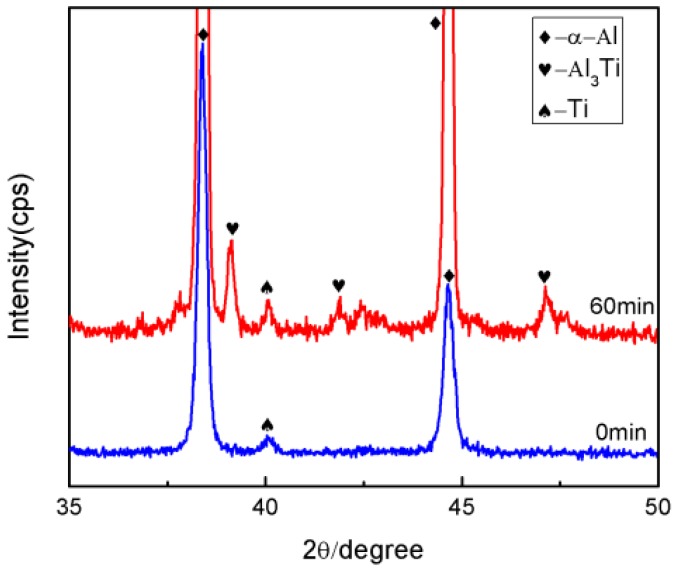
An XRD spectrum of the mixed powder bulk heated for 0 min and 60 min at 640 °C.

During the partial remelting process, a diffusion reaction can occur at the Al melt/Ti interface. The diffusion coefficients for aluminum in solid titanium (*D_Al/Ti_*) [21] and for solid titanium in liquid aluminum (*D_Ti/Al_*) [22] can be calculated using the following equations:

(1) $$D_{Al/Ti}=9.58\times 10^{-9}m^{2}/s\times \exp\left[\frac{-114600J/mol}{8.31J/molK\cdot T}\right]$$

(2) $$D_{Ti/Al}=1.12\times 10^{-1}m^{2}/s\times \exp\left[\frac{-260000J/mol}{8.31J/molK\cdot T}\right]$$

where the *D_Al/Ti_* is the diffusion coefficients for aluminum in solid titanium; the *D_Ti/Al_* is the diffusion cofficients for solid Ti in liquid aluminum; and *T* is the thermodynamic temperature. When the heating temperature is *T* = 913 K (640 °C), the values of *D_Al/Ti_* [21] and *D_Ti/Al_* [22] are equal to 2.64 × 10^−15^ m^2^/s and 1.47 × 10^−16^ m^2^/s, respectively. Thus, the solubility of Al in Ti is greater than the solubility of Ti in Al [23]. Therefore, Al is the main diffusion component in the Al-Ti diffusion couple. The initial diffusion of the Al atoms contacting the solid Ti particles through the Ti/Al interface can produce a saturated solution adjacent to the interface, resulting in the nucleation of the Al_3_Ti phase on the solid titanium powder surface at the Ti/Al interface. An Al_3_Ti phase rather than AlTi_3_ and AlTi phases is preferentially formed in the Al(l)-Ti(s) system due to its lower free energy of formation [24] and a rich Al environment. Figure 9 depicts the high magnification SEM micrographs of the mixed powder bulk heated at 640 °C for different time lengths. Figure 9a shows the initial microstructures of the cold-pressed ingot. After being cold pressed, the Ti particles mechanically consolidate with the matrix alloy powders. The initial diffusion reaction can be expressed as 4Al + 3TiO_2_ = 3Ti +2Al_2_O_3_, 3Al +Ti = Al_3_Ti due to the TiO_2_ oxide film existing on the Ti powder surface. When all TiO_2_ is completely consumed, the molten Al begins to react with the Ti powder continuously. When the heating time reaches 5 min, the reaction between Al and TiO_2_ occurs at the interface due to the diffusion of Al atoms (Figure 9b). On the other hand, the point scanning results indicate that the Ti atoms diffuse into the Al matrix (Figure 10 and Table 3). Thus, the reaction between Al and Ti is an interdiffusion reaction. As the heating time increases, a small Al_3_Ti phase is formed on the Ti powder surface due to the rapid temperature increase and the formation of the liquid films required for the reaction (Figure 9c). Figure 11 shows the XRD pattern of the studied sample illustrating the formation of the Al_3_Ti phase. In addition, some weak Ti peaks are present, suggesting that the Ti powder did not react completely due to a short heating time. After heating for 15 min, the amount of the liquid phase continuously increases, and the atomic diffusion rate accelerates due to the temperature increase. Furthermore, the diffusion rate of the Al atoms in Al_3_Ti is higher than the diffusion rate of Ti atoms, so the Al atoms can spread in the interior of the Ti particles by forming an Al_3_Ti layer. As a result, the Al_3_Ti reaction layer can go inward [23], and a relatively thin, dense reaction layer is formed on the titanium powder surface (Figure 9d). In the subsequent reaction process, the thickness of the reaction layer continuously increases with heating time. However, when the thickness reaches a certain threshold at a given size of the Ti powder, cracks and pores can appear inside the reaction layer (Figure 9e). This phenomenon can have two explanations. First, the Kirkendall effect caused by the difference in *D_Al/Ti_* [21] and *D_Ti/Al_* [22] results in a pore formation. Second, the pores can be produced in the reaction layer [11] due to the stress caused by the volume expansion during the period of the Ti transformation into Al_3_Ti.

The volume change during the reaction can be expressed as

(3) $$\Delta V=\frac{\sum V_{products}-\sum V_{reac\tan ts}}{\sum V_{reac\tan ts}}$$

where the ΔV is the volume change during the reaction; the $\sum V_{products}$ is the total atomic volume of products; the $\sum V_{reac\tan ts}$ is the total atomic volume of reactants. The volume of dilatation during the transformation of Ti into Al_3_Ti can be calculated by using Equation (3) [25]:

(4) $$\Delta V=\frac{V_{Al_{3}Ti}-V_{Ti}}{V_{Ti}}$$

where $V_{Al_{3}Ti}$ is the atomic volume of the Al_3_Ti compound; and $V_{Ti}$ is the atomic volume of the titanium. The corresponding values of $V_{Al_{3}Ti}$ and $V_{Ti}$ are shown in the Table 2.

By calculating, the obtained value of Δ*V* is 2.61, which means that the volume dilatation of the Ti particles after reacting with molten Al is about 261%.

Simultaneously, the stress in the reaction layer can be calculated using Equation (5) [25]:

(5) $$\sigma_{Al_{3}Ti}=-\frac{E_{Al_{3}Ti}}{6\left(1-\upsilon_{Al_{3}Ti}\right)}\frac{{t^{2}}_{Al_{3}Ti}}{t_{Ti}}\left(\frac{1}{R}-\frac{1}{R_{0}}\right)$$

where $\sigma_{Al_{3}Ti}$ is the stress in the Al_3_Ti reaction layer; E, υ are the modulus of elasticity and the Poisson’s ratio of Al_3_Ti; $t_{Al_{3}Ti}$ and $t_{Ti}$ are the thicknesses of the Al_3_Ti reaction layer and the residual Ti after the reaction with the molten Al, respectively; *R* and *R_0_* are the radii of the Ti particles after and before the reaction, respectively.

The measurement results indicate that the maximum thickness of the Al_3_Ti reaction layer before it peels off from the Ti phase surface is about 1.63 μm, and $t_{Ti}$ is equal to about 8.85 μm. *R* is equal to 10.26 μm for the Ti powder with a particle size of around 9.28 μm. The calculated value of stress is about 15.89 GPa, which is larger than the theoretical fracture strength of Al_3_Ti (14.4 GPa) [26]. Therefore, it is possible for the microcracks to appear in the reaction layer and for the layer itself to peel off from the Ti phase surface when the thickness of the reaction layer exceeds a certain threshold.

**Figure 12:**
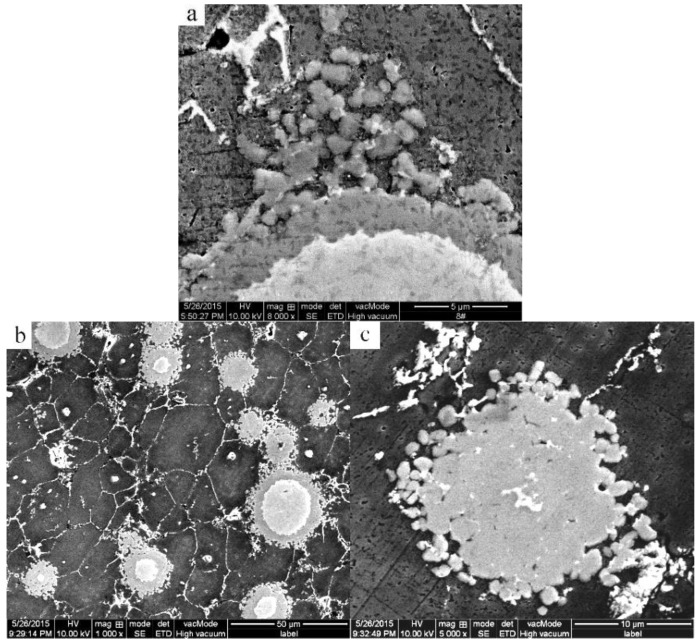
High magnification SEM micrographs of the mixed powder bulk heated at 640 °C for (**a**) 90 min; (**b**) 210 min; (**c**) 210 min.

**Figure 13:**
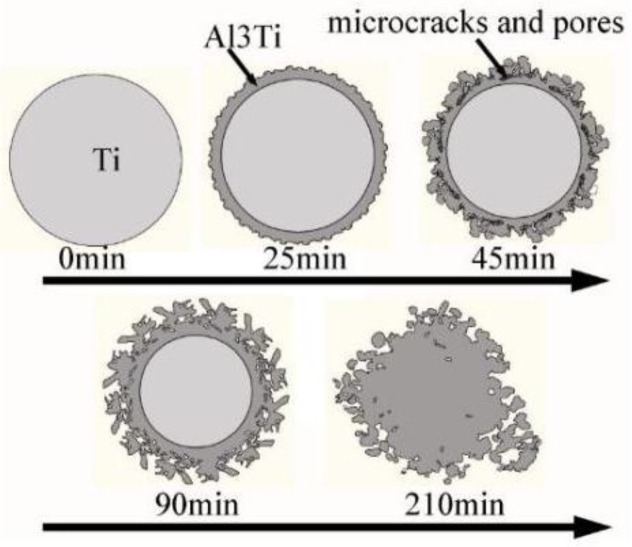
An illustration of the Al_3_Ti phase formation process.

With the heating time increased from 45 to 90 min, the reaction layer peels off from the Ti phase surface in a form of small particles due to the release of the tensile stress and brittleness of the Al_3_Ti compound. Subsequently, the small peeled Al_3_Ti particles migrate away into the aluminum matrix and coarsen (comparing Figure 9e,f) due to the Ti atoms’ diffusion into the surrounding of the Al_3_Ti particles through the grain boundaries. Furthermore, the rupture of the Al_3_Ti layer enables the residual Ti to first contact, and then react with the Al liquid, thus eventually transforming into the Al_3_Ti particles at longer heating times (Figure 12b,c). It is clearly seen from Figure 12c that the reaction layer does not completely break into the Al_3_Ti particles except for the outermost surface layer in order to reduce the interfacial energy between the Al_3_Ti phase and the molten Al. The whole formation process of the Al_3_Ti phase is illustrated in Figure 13.

### 3.4. Analysis of the Simulation Experiment Results for the Formation of the Al_3_Ti Phase

To study the evolution and growth behavior of the Al/Ti interface microstructure, molten aluminum was dripped into a 10 mm × 10 mm pure titanium plate to form a sample. The sample was then placed inside a stainless crucible. The crucible with the sample was in turn placed into a vacuum furnace and reheated at a semisolid temperature of the 2024 aluminum alloy (640 °C) for 1, 2, 3, 5, and 8 h. After the sample was treated, ground, and polished, the bonding situations and microstructures were observed under a scanning electron microscope.

**Figure 14:**
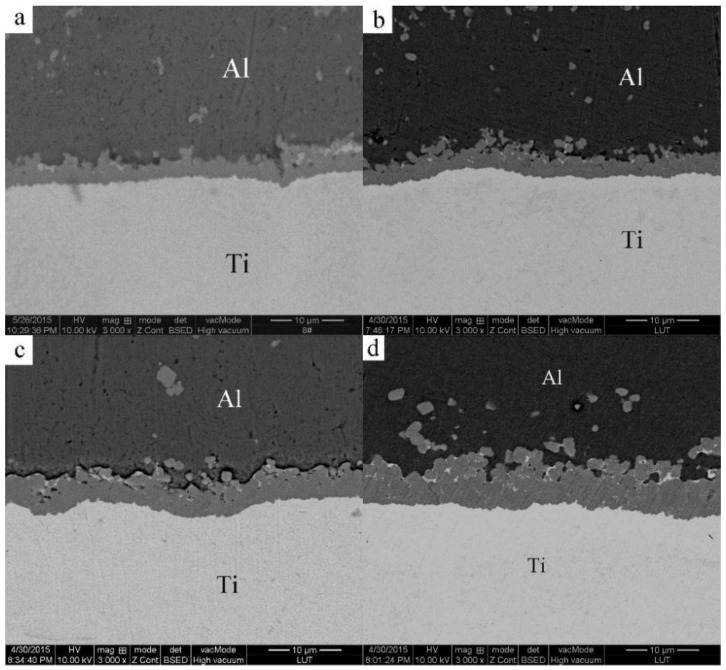

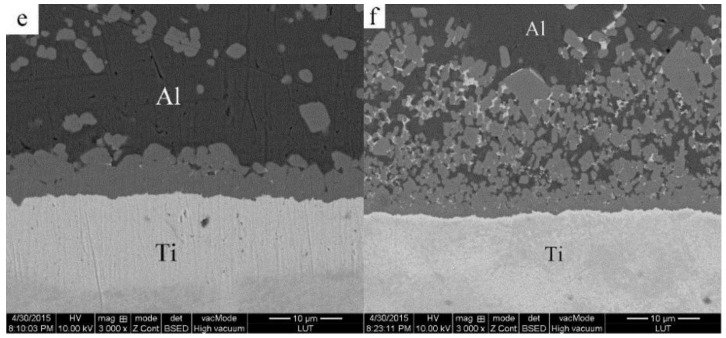
SEM micrographs of the Al-Ti interface after heating at 640 °C for different lengths of time: (**a**) 1 h; (**b**) 2 h; (**c**) 3 h; (**d**) 5 h; (**e**) 8 h; (**f**) 15 h.

**Figure 15:**
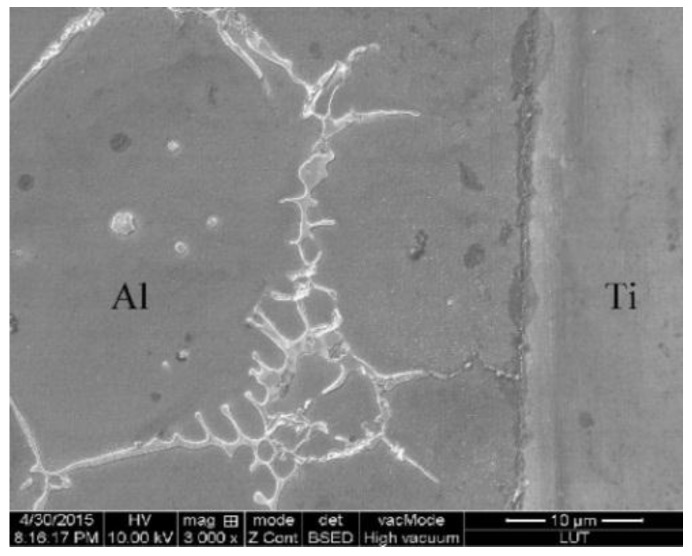
SEM micrographs of the Al-Ti liquid-solid interface after heating at 640 °C for 1 h and then etching by the Keller solution.

**Figure 16:**
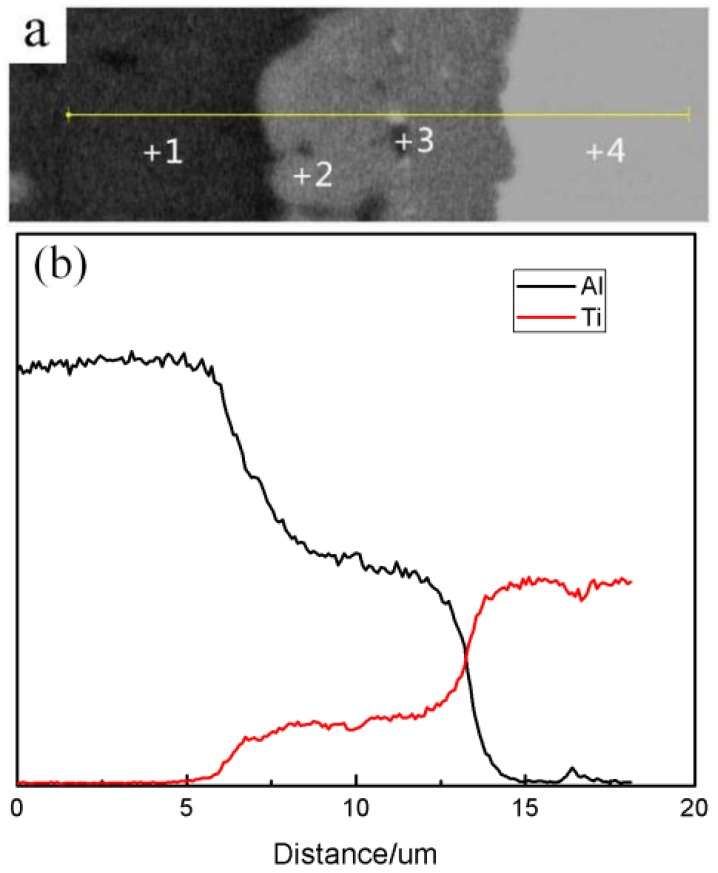
An SEM line and point scan analysis of the sample remelted at 640 °C for 8 h. (**a**) SEM micrograph of line and point scan; (**b**) Data analysis of line scan.

**Figure 17:**
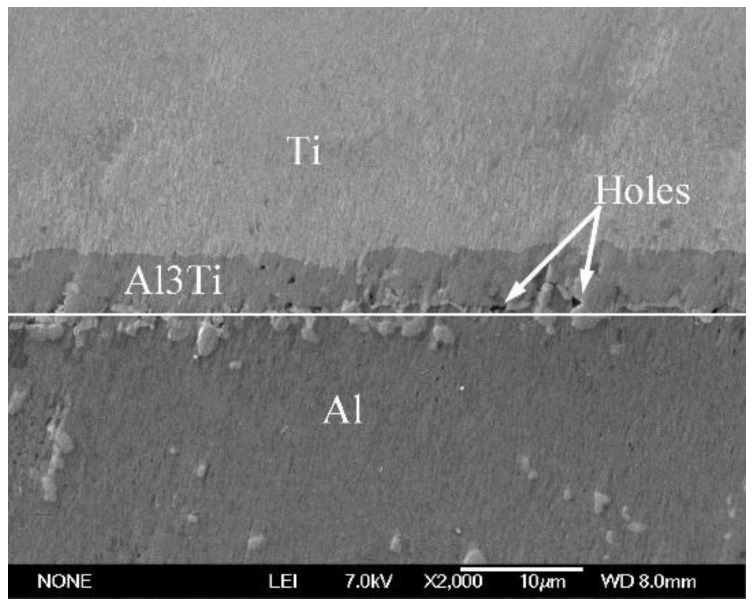
An SEM micrograph of the Al/Ti interface after heating for 5 h.

Figure 14 shows the SEM images of the Al/Ti diffusion couples, which were heated at 640 °C for different lengths of time. After heating for 1 h, a relatively thin reaction layer was detected at the Al/Ti interface. With an increase in the heating time, the thickness of the reaction layer and the amount and size of the individual particles outside the diffusion reaction zone peeled from the Al_3_Ti reaction layer increase as well. Furthermore, the individual particles move towards the Al alloy matrix, possibly due to the difference in the ratio of gravity between the Al_3_Ti and molten Al, and the flowing liquid metal. On the other hand, an appearance of the liquid phase is observed at the grain boundaries of the aluminum alloy after etching with the Keller solution (Figure 15). To further confirm the microstructure of the reaction layer at the Al/Ti interface, a sample remelted at 640 °C for 8 h was investigated by EDS. The corresponding point positions and line scanning curves are depicted in Figure 16a and b, respectively. According to the line-scanning spectrum, the Ti and Al compositions vary at the interface between the reaction layer and Ti/Al indicating the formation of an intermetallic compound moving towards the Al and Ti phases simultaneously (Figure 17). The point spectrum data demonstrates that the Al contents at points 1, 2, 3, and 4 are 98.6%, 75.2%, 92.8% and 0, respectively (Table 4). After analysis, it can be easily seen that the compositions of points 1, 2, and 4 are Al, Al_3_Ti, and Ti, respectively (Table 4). Therefore, the microstructure of the Al/Ti diffusion couple can be described as Al/(Al_3_Ti + Al)/Ti (at the given experimental conditions).

The reason behind the microstructure formation for the Al/Ti diffusion couple can be described as follows. The essence of the reaction studied in this work is interdiffusion (Figure 10). However, the solubility of Ti at the given conditions is extremely low (about 1% in molten aluminum at 900 °C) [31]. The Ti atoms saturate soon after they diffuse into the molten Al, and then the oversaturated Ti atoms react with the Al atoms to form the solid Al_3_Ti. In the Al/Ti phase diagram [31], there is an area of coexistence between the solid Al_3_Ti and molten aluminum, so Al_3_Ti can nucleate in the oversaturated molten Al and continue dissolving and diffusing Ti atoms until the Al_3_Ti and molten Al reach their chemical equilibrium. Therefore, the microstructure of the reaction layer at the Al/Ti interface is a mixed structure of Al and gray particles of Al_3_Ti. With increasing heating time, the Ti phase continues to decompose, diffuse, and react, thus increasing the thickness of the reaction layer.

**Figure 18:**
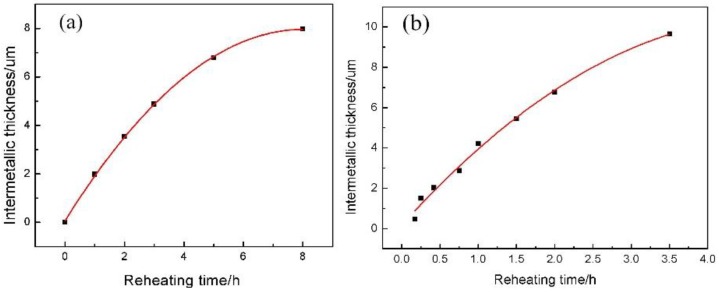
(**a**) An evolution of the average total intermetallic sample thickness during the remelting process; (**b**) A reaction layer thickness dependent on the heating time after heating for 10 min at 640 °C.

To further investigate the growth mechanism for intermetallic compounds, the total intermetallic thickness is plotted against the reaction time in Figure 18a. The measured layer thickness *x vs.* time *t* can be modeled with an empirical power law relationship provided by Equation (6) [32,33]:

(6) $$x=x_{0}+A{\left(t\right)}^{n}$$

where $x_{0}$ is the thickness of the reaction layer at t=0; A is the growth constant; and n is the time exponent. The values of the time exponent n can be obtained by a multivariable linear regression analysis corresponding to Equation (7) [33]:

(7) $$\ln\left(x-x_{0}\right)=\ln A+n\ln t$$

Here the time exponent values are obtained from the slope of the plot of $\text{ln}\left(x-x_{0}\right)$ *versus* ln(t).

Three different time exponents were obtained from the calculation results for the whole reaction process, which can be used to characterize the measured growth rate. This implies that different growth mechanisms exist at *n* = 0.58 and 1–2 h of reaction time, *n* = 0.165 and 2–5 h of reaction time, and at *n* = 0.105 and 5–8 h of reaction time. In the early stages, the initial interdiffusion of the Ti and Al atoms across the Al/Ti interface can produce an adjacent saturated solution, resulting in the nucleation of the Al_3_Ti phase on the solid Ti surface at the Al/Ti interface and a rapid formation of a planar Al_3_Ti intermetallic layer with significant thickness, as observed in Figure 15. The initial intermetallic thickness likely corresponds to the interdiffusion distance between the Al and Ti atoms in the initial intermetallic layer. After 1–2 h of the reaction with the time exponent *n* = 0.58, the growth rate is high, probably due to the short diffusion distance. After 2–5 h of the reaction, the time exponent is *n* = 0.165. During this stage of the reaction, the intermetallic growth is dominated by the grain boundary diffusion [34], and the reaction layer consists of many grains, whose boundaries are likely to provide a diffusion path for the Al atoms to move into the solid Ti. On the other hand, the formed reaction layer provides a barrier to diffusion. Therefore, the diffusion becomes slower compared to the initial stage. After 5 h of the reaction with the time exponent *n* = 0.105, the rate of intermetallic growth is slowed again. The Al_3_Ti phase growth mechanism should still be dominated by the grain boundary diffusion, but the diffusion distance is longer compared with the first two stages. From the results of this experiment, it is expected that the intermetallic formation and growth mechanism of the reaction between Al and Ti are controlled by the nucleation barrier and the diffusion distance followed by the grain boundary diffusion of the Al atoms into the solid Ti.

Very little difference is observed when these two experimental results are compared (see Figure 18a,b). 

At the value of *n* equal 2, the growth rate of the reaction layer obeys the similarity law in both diffusion experiments and can be expressed as

(8) $$X=At^{2}+Bt+C$$

where *X* is the zone thickness; *A*, *B* and *C* are the growth rate constants, and *t* is the diffusion time. Deviations from this law can be explained by the grain boundary diffusion or interface reaction-controlled growth. Therefore, this experiment can be a good simulation of the interfacial reaction between the Ti and Al powders, which allows estimation of the variations in the reaction layer thickness with the heating time more accurately. From the simulation data, the following expressions were obtained:

(9) $$X_{a}=-0.12t^{2}+1.97t+0.06$$

(10) $$X_{b}=-0.43t^{2}+4.21t+0.17$$

Equation (9) can be used to characterize the reaction rate for the mixture of the Ti plate and Al alloy, while Equation (10) describes the reaction rate for the mixture of the Ti and Al powders. Comparing these two equations, it is clearly observed that the reaction rate of the Ti and Al powders is greater because $\left|A_{b}\right|=0.43>\left|A_{a}\right|=0.12$. On the other hand, the interface between the Ti powder and the liquid phase is larger than the interface between the Ti plate and the liquid phase (most likely due to the spherical shape of the Ti particles).

## 4. Conclusions

(1) A semisolid microstructure with small spheroidal particles was obtained after the mixed powder bulk was remelted at 640 °C. The growth of the Al_3_Ti reaction layer during the remelting process follows a parabolic law $X=-0.43t^{2}+4.21t+0.17$.

(2) Compared with the experimental results of the drip experiment, the reaction rate for the mixture of the Ti powder and the Al powder was faster than the reaction rate for the mixture of the Ti plate and the Al alloy, respectively.

(3) During partial remelting, the microstructural evolution of the 2024 alloy matrix can be divided into three stages: a rapid coarsening of powder grains due to the dissolution of the intergranular eutectics (0–5 min), a formation of spherical primary α-Al particles surrounded by a continuous liquid film due to the rapid temperature increase (5–15 min), and a slight coarsening of the spherical primary α-Al particles in order to reduce the interfacial energy (after 15 min).

(4) An Al_3_Ti reaction layer is formed on the surface of the Ti powder by the interdiffusion of the Ti and Al atoms, while the reaction layer moves inward in the radial direction. When the layer thickness increases to a certain threshold for the Ti powder with a particular particle size, cracks can form, and the layer peels off from the Ti phase surface in a form of small particles.

(5) The stress caused by the volume dilatation during the transformation of Ti into Al_3_Ti can be calculated by using the following equations: $\Delta V=\frac{V_{Al_{3}Ti}-V_{Ti}}{V_{Ti}}$ and $\sigma_{Al_{3}Ti}=-\frac{E_{Al_{3}Ti}}{6\left(1-\upsilon_{Al_{3}Ti}\right)}\frac{{t^{2}}_{Al_{3}Ti}}{t_{Ti}}\left(\frac{1}{R}-\frac{1}{R_{0}}\right)$.

## Figures and Tables

**Table 1:** Compositions of different structures of the bulk alloy heated at 640 °C for different lengths of time.

Heating Time/min	Structure	Composition (wt.%)		
		Al	Cu	Mg
0	Pure aluminum	100	0	0
	2024 aluminum	90.2	8.3	1.5
5	Pure aluminum	97.4	2.1	0.5
	2024 aluminum	94.1	4.8	1.1
10	Pure aluminum	94.3	4.8	0.8
	2024 aluminum	94.2	5.0	0.8

**Table 2:** Molar volume and elastic modulus of Al, Ti (for the bulk material at room temperature), and the TiAl_3_ alloy [27,28,29,30].

Material	Molar Volume $cm^{3}/mole$	Elastic Modulus $\frac{E}{\left(1-\upsilon \right)}\left(10^{5}MPa\right)$
Al	9.995	1.143
Ti	10.629	1.699
TiAl_3_	38.408	3.086

**Table 3:** Compositions of the points shown in Figure 10.

Point	Composition (wt.%)			
	Al	Ti	Mg	Cu
1	63.0	35.6	0.5	0
2	75.2	24.6	0.2	0
3	88.3	11.3	0.4	0
4	92.1	7.8	0.1	0

**Table 4:** Compositions of the sample points after remelting at 640 °C for 8 h.

Point	Composition (wt.%)		Phase
	Al	Ti	
1	98.6	0	Al
2	75.2	24.6	Al_3_Ti
3	92.8	5.8	(Al)
4	0	99.4	Ti
